# Inverted U-Shaped Association Between Serum Uric Acid and Handgrip Strength in Chinese Community-Dwelling Adults: A Repeated-Measures Cohort Study

**DOI:** 10.3390/biomedicines14030568

**Published:** 2026-03-02

**Authors:** Rui Ma, Yujia Ma, Xiaoyi Li, Kexin Ding, Han Xiao, Yan Liu, Dafang Chen, Wei Cao

**Affiliations:** 1 Department of Rheumatology, Wang Jing Hospital of China Academy of Chinese Medical Sciences, Beijing 100102, China; 15765288780@163.com; 2 Wang Jing Hospital, Beijing University of Chinese Medicine, Beijing 100029, China; 3 Department of Non-Communicable Chronic Disease Control and Prevention, Beijing Center for Disease Prevention and Control, Beijing 100013, China; yujiama@pku.edu.cn; 4 Department of Epidemiology and Health Statistics, School of Public Health, Capital Medical University, Beijing 100069, China; 5 Department of Epidemiology and Biostatistics, School of Public Health, Peking University, Beijing 100191, China; 6 Yingdong Intelligent Technology (Shandong) Co., Ltd., Jinan 250102, China; 7 Key Laboratory of Epidemiology of Major Diseases (Peking University), Ministry of Education, Beijing 100191, China

**Keywords:** serum uric acid, handgrip strength, sarcopenia, restricted cubic spline, cohort study

## Abstract

**Background:** Handgrip strength (HGS) is a robust indicator of muscle strength and overall health, yet its relationship with serum uric acid (SUA) remains unclear due to limitations in linear modeling. This study investigated the nonlinear relationship between SUA and HGS using repeated-measures cohort data. **Methods:** The cohort consisted of 3016 community-dwelling Chinese adults contributing 4439 repeated measurements (2021–2024). SUA was measured via the uricase–peroxidase method, and HGS was measured via digital dynamometry. Nonlinear associations were evaluated using restricted cubic spline (RCS) linear mixed-effects models and subgroup and sensitivity analyses. **Results:** No significant linear association was found (*p* = 0.170). However, RCS analysis revealed an inverted U-shaped curve of the peak HGS at approximately 404 μmol/L SUA (*p* for nonlinearity < 0.05). When SUA ≤ 404 μmol/L, each standard deviation increase in SUA was associated with a 1.74 kg increase in HGS (*p* = 0.003), and when SUA > 404 μmol/L, each standard deviation elevation in SUA corresponded to a 0.57 kg decrease in HGS (*p* = 0.049). This inverted U-shaped relationship was significant in men and participants without type 2 diabetes mellitus (T2DM) but was not observed in women (*p* for interaction < 0.05) or T2DM patients. Sensitivity analyses confirmed the validity of these results. **Conclusions:** An inverted U-shaped relationship between SUA and HGS was observed in Chinese adults with an inflection point at approximately 404 μmol/L, modified by sex and notably observed in non-T2DM participants. The results suggest that both low and high SUA levels may impair muscle function, highlighting SUA’s potential as a biomarker for muscle decline and the importance of population-specific management strategies.

## 1. Introduction

Muscle strength, assessed through handgrip strength (HGS) measurement, represents a critical biomarker for evaluating individual health status and the progression of biological aging [1]. Within sarcopenia diagnostic frameworks, reduced HGS serves as a direct indicator of age-associated decline in muscle mass and functional capacity while also functioning as a prospective marker for adverse clinical outcomes such as cancer, cardiovascular disease, and all-cause mortality [2,3,4,5,6]. The robust predictive capacity of HGS, combined with its simplicity of measurement, underscores the clinical and public health importance of identifying modifiable factors that impact this parameter to delay muscle senescence and enhance quality of life in aging populations.

Emerging research has highlighted serum uric acid (SUA) as a potentially valuable, albeit controversial, biomarker linked to musculoskeletal health. The existing literature reveals inconsistent patterns regarding the SUA–HGS relationship. Certain observational investigations have identified that SUA has muscle-protective properties, documenting positive correlations with HGS [7,8,9]. In contrast, alternative findings indicate that higher SUA concentrations may compromise muscle integrity, correlating with elevated sarcopenia risk and progressive muscle depletion [10,11,12]. Such contradictory results likely stem from SUA’s paradoxical biological behavior, as it exerts variable effects on muscle tissue depending on its concentration [13]. Within physiological ranges, SUA demonstrates extracellular antioxidant activity [14,15]; conversely, excessive concentrations trigger pro-oxidant mechanisms [16,17,18,19]. This concentration-dependent duality implies a potentially nonlinear SUA-HGS association. Recent cross-sectional investigations have provided preliminary evidence of an inverted J-shaped pattern characterizing this relationship [20,21]. Despite revealing nonlinear trends, these studies face inherent limitations due to their single-assessment design, which cannot elucidate temporal dynamics in the dose–response relationship. Consequently, longitudinal cohort investigations incorporating repeated assessments and employing sophisticated nonlinear analytical approaches—particularly restricted cubic spline (RCS) modeling—are crucial for precisely delineating the SUA-HGS association and determining critical physiological thresholds for SUA.

Currently, there is a paucity of longitudinal data utilizing repeated measurements to establish SUA’s effects on muscle strength. Addressing this knowledge gap, our investigation leveraged serial measurement data from a Chinese adult population cohort, implementing an RCS methodology to examine the nonlinear dose–response pattern linking SUA to HGS and to delineate potential optimal SUA concentration ranges. Our results offer evidence-based guidance for metabolic management interventions designed to maintain muscle functional capacity.

## 2. Materials and Methods

### 2.1. Study Population

This study focuses on a community-based prospective cohort using a mobile health (mHealth) application [22]. The cohort was recruited and followed by community-dwelling adults in five provinces (Anhui, Guangdong, Shandong, Shanxi, and Tianjin) in China through a commercial mHealth application, “YIDO Health” (YIDO Artificial Intelligence Technology (Shandong) Co., Ltd., Jinan, China), with consumer-grade wearable devices [22]. This application allows for personal health monitoring, assessment, and guidance via digital technology. Users connect their wearable devices to smartphones and upload the monitored data to an online platform. We used de-identified data from this platform without direct personal information. Instead, a random string was used as the participant ID, linking different measurement records. Participants were included based on the following criteria: (1) age ≥ 18 years; (2) availability of paired SUA and HGS data for at least one year between 2021 and 2024; and (3) electronic informed consent obtained before participation. The exclusion criteria were (1) missing baseline body mass index (BMI) data and (2) more than 20% missing data for other covariates.

**Figure 1:**
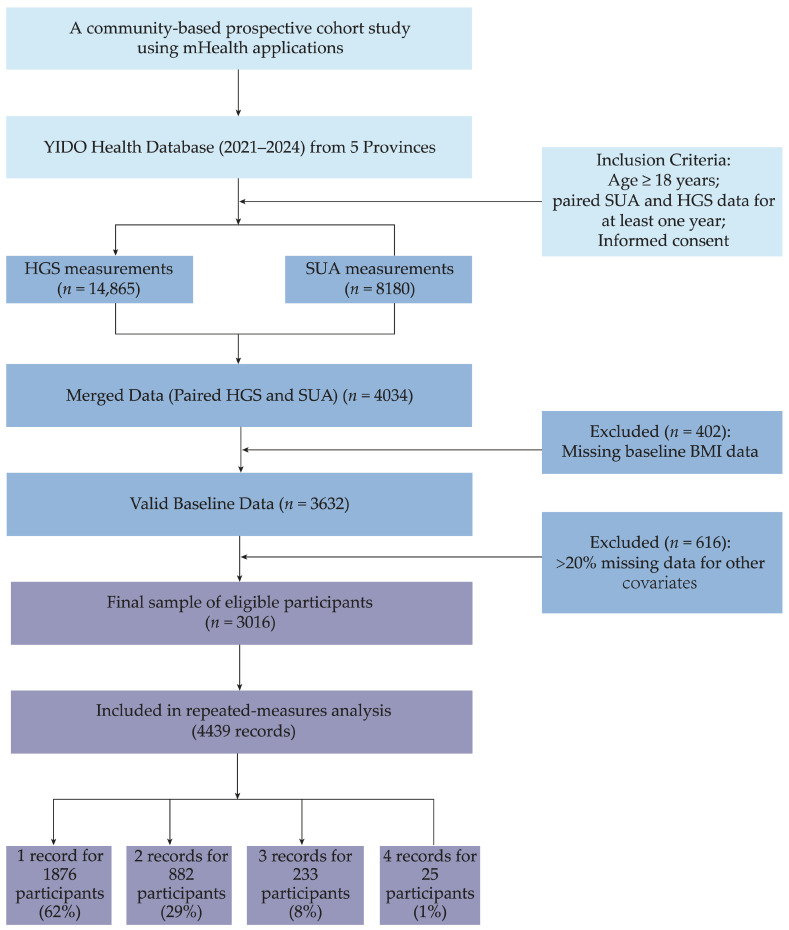
Flowchart of participant selection created using Figdraw. Abbreviations: BMI, body mass index; HGS, handgrip strength; SUA, serum uric acid.

The screening process is shown in Figure 1. First, we identified participants with HGS measurements (N = 14,865) and SUA measurements (N = 8180) in the 2021–2024 period, finding 4034 participants with both types of data. We then screened for data integrity, where 402 participants lacking BMI data were excluded, and an additional 616 participants with over 20% missing data for other covariates were also removed. Ultimately, 3016 eligible participants were included in the 4439 repeated measures. The study protocol received approval from Biomedical Ethics Committee of Peking University (PU IRB) (No. IRB00001052-23179). All participants provided electronic informed consent via their YIDO accounts, which allowed us to use their anonymized data for research purposes.

### 2.2. Measurement of SUA

Trained medical staff collected fasting antecubital vein blood samples from participants who had fasted for more than 8 h. Serum biochemical parameters were measured using standard procedures at the hospital’s clinical laboratory. The fasting SUA concentration was measured using the uricase–peroxidase method on an ADVIA 2400 automated biochemical analyzer (Siemens AG, München, Germany) with a detection range from 0 μmol/L to 1190 μmol/L. Intra-assay and inter-assay coefficients of variation (CV) were less than 2% and 3% [23].

### 2.3. Measurement of HGS

HGS was measured with a standard digital hand dynamometer (LM—G001, Qingdong Technology (Shenzhen) Co., Ltd., Shenzhen, China) in kilograms (kg). The device was connected wirelessly to a tablet application to perform testing and automatically record data. The participants stood upright, with their arms hanging naturally at their sides without touching the body, and squeezed the dynamometer with maximum force for at least 6 s. Each participant performed two trials on each hand, with a 60 s rest interval between trials. The maximum of the four measurements (two trials for each hand) was recorded as the final HGS value (kg) [24].

### 2.4. Aggregation of SUA and HGS Measurements

SUA and HGS were analyzed annually. For each participant, all available SUA and HGS measurements obtained within the same calendar year were paired and aggregated, and the annual mean value was used as the representative measurement for that year. Although SUA and HGS measurements were not necessarily collected on the same day, restricting the pairing within the same year ensured temporal proximity between exposure and outcome at the annual level. This approach reduces random within-year measurement variability and minimizes the impact of short-term biological variation.

### 2.5. Assessment of Covariates

The covariates included demographic characteristics, anthropometric and lifestyle factors, and baseline medical history [25]. Demographic characteristics included age (years), gender (male/female), geographical region (province), and educational level (primary school or below, junior high school, senior high school, and college or above). Anthropometric measurements included BMI, which was calculated as weight (kg) divided by the square of height (m) (kg/m^2^). Regarding lifestyle factors, regular physical activity was self-reported and categorized as a binary variable (yes/no). Baseline medical history, including hypertension, T2DM, and hyperlipidemia, was confirmed from self-reported doctor-diagnosed history and laboratory tests. Hypertension was defined as mean systolic blood pressure ≥ 140 mmHg, mean diastolic blood pressure ≥ 90 mmHg, self-reported doctor diagnosed history, or current use of antihypertensive drugs [26]. T2DM was determined if any of the following criteria were met: glycated hemoglobin (HbA1c) > 6.5%; fasting blood glucose ≥ 7.0 mmol/L; random blood glucose ≥ 11.1 mmol/L; 2 h blood glucose ≥ 11.1 mmol/L in the oral glucose tolerance test (OGTT); self-reported doctor-diagnosed history; or current use of hypoglycemic drugs [27]. Hyperlipidemia was defined as one or more of the following conditions: serum total cholesterol ≥ 5.17 mmol/L; triglycerides ≥ 1.69 mmol/L; low-density lipoprotein cholesterol ≥ 3.62 mmol/L; high-density lipoprotein cholesterol ≤ 1.04 mmol/L; self-reported doctor-diagnosed history; or current use of lipid-lowering drugs [28].

### 2.6. Statistical Analysis

We summarized the baseline characteristics of the study population at the first entry. Continuous variables are reported as the mean (standard deviation, SD) and median (interquartile range, IQR), according to the data distribution. The categorical variable is reported as frequency (percentage). We used the *t*-test, Mann–Whitney U test, or chi-square test for group comparisons.

#### 2.6.1. Missing Data Processing

We dealt with missing data as follows. First, we excluded populations without BMI information. For other covariates, we calculated the missing data rate for each individual and study population with more than 20% missing covariates. Lastly, we imputed variables with a missing rate of less than 5% using the mean for continuous variables and the mode for categorical ones.

#### 2.6.2. Analysis of the Association Between SUA and HGS

We used a linear mixed-effects model (LMM) to assess the relationship between SUA and HGS with the repeated measurements of the population. This method accommodates individual correlations in repeated observations and effectively handles unbalanced data due to different measurement counts. We evaluated the linear association using three sequential multivariable models. Model 1 was adjusted for age, gender, and region. Model 2 added adjustments for BMI, education level, and physical activity to Model 1. Model 3 was adjusted for T2DM, hypertension, and hyperlipidemia based on Model 2.

#### 2.6.3. Nonlinear Correlation Analysis

We tested for a possible nonlinear correlation using RCS with three knots in an LMM. The knots were set at the 10th, 50th, and 90th percentiles to flexibly model the dose–response curve. We used the likelihood ratio test to determine whether the nonlinear model fit better than the linear model. Based on the inflection points from the RCS analysis, we performed a segmented analysis. This involved fitting the LMM separately for participants with SUA levels below and above the threshold, allowing us to measure the association strength between SUA and HGS on each side of the threshold. The covariates adjusted in the model matched those in Model 3.

#### 2.6.4. Subgroup Analysis and Sensitivity Analysis

We performed stratified analyses, refitting the full nonlinear and segmented models for each subgroup. To verify the robustness of the results, we conducted sensitivity analysis using the LMM and compared the main results. The *p* values for interaction were obtained from joint Wald tests of all RCS-based interaction terms between serum uric acid and each subgroup variable.

We performed all statistical analyses using R (version 4.0.0), and all analyses were statistically significant with a two-sided *p* value < 0.05.

## 3. Results

### 3.1. Study Population and Baseline Characteristics

This study enrolled 3016 participants at baseline, resulting in 4439 records. Of these participants, 62% (1876/3016) provided one record over 4 years, 29% (882/3016) provided two records, 8% (233/3016) provided three records, and 1% (25/3016) provided four records. The cohort comprised 2045 females (67.8%) and 971 males (32.2%). Table 1 details the baseline characteristics by gender. Males had a significantly higher mean age than females (*p* < 0.001). Significant differences also appeared in regional distribution (*p* = 0.001) and educational level (*p* < 0.001), with more males in the group with the highest educational level. No significant difference was found in BMI between genders. Hypertension and T2DM were significantly more prevalent in males (*p* < 0.001), while hyperlipidemia was significantly more prevalent in females (*p* < 0.001). Males also had significantly higher mean SUA levels and HGS compared to females (*p* < 0.001).

### 3.2. Association Between SUA and HGS

**Figure 2:**
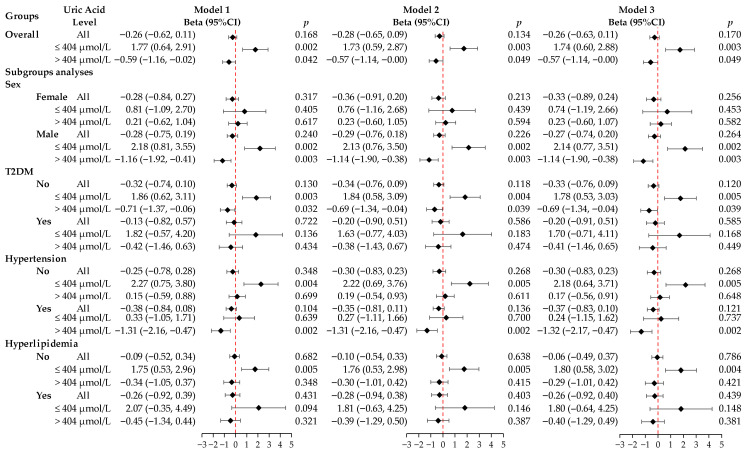
Forest plot of beta coefficients and 95% CIs for the association of serum uric acid with handgrip strength. Results are derived from linear mixed-effects models for both the overall linear trend and a two-piecewise segmented analysis with a threshold of 404 μmol/L. The models were sequentially adjusted as follows: Model 1 was adjusted for age, gender, and region; Model 2 added adjustments for BMI, education level, and regular physical activity to Model 1; and Model 3, the fully adjusted model, was adjusted for T2DM, hypertension, and hyperlipidemia based on Model 2. Abbreviations: CI, confidence interval; T2DM, type 2 diabetes mellitus; BMI, body mass index.

In our initial LMM analysis, we found no significant overall linear relationship between SUA and HGS in the fully adjusted model (Model 3) (β = −0.26, 95% CI: −0.63–0.11, *p* = 0.170). However, subsequent RCS analysis revealed a significant nonlinear inverted U-shaped association between SUA and HGS (nonlinear *p* value < 0.05), with the highest HGS observed at an SUA level of approximately 404 µmol/L. In Model 3, the segmented analysis showed that when the SUA level was ≤404 µmol/L, for every one standard deviation increase in SUA, HGS significantly increased by 1.74 kg (95% CI: 0.60–2.88, *p* = 0.003). Conversely, when the SUA level was >404 µmol/L, for every one standard deviation increase in SUA, HGS decreased by 0.57 kg (95% CI: −1.14–−0.00, *p* = 0.049) (Figure 2).

### 3.3. Subgroup Analysis

We further performed subgroup analyses based on gender, T2DM, hypertension, and hyperlipidemia (Figure 2). The inverted U-shaped relationship between SUA and HGS was most pronounced in men. In men, SUA levels ≤ 404 µmol/L correlated with increased HGS (β = 2.14, 95% CI: 0.77–3.51, *p* = 0.002), while SUA levels > 404 µmol/L correlated with decreased HGS (β = −1.14, 95% CI: −1.90–−0.38, *p* = 0.003). No significant link was found in women. Similarly, this association was significant in non-T2DM participants. SUA levels ≤ 404 µmol/L were linked to increased HGS (β = 1.78, 95% CI: 0.53–3.03, *p* = 0.005), whereas levels > 404 µmol/L were linked to decreased HGS (β = −0.69, 95% CI: −1.34–−0.04, *p* = 0.039). However, no significant association was observed in patients with T2DM. When RCS-based interaction terms were introduced into the linear mixed-effects models, statistically significant nonlinear interactions were observed between SUA and sex (*p* for interaction < 0.05), whereas no significant interactions were found for T2DM, hypertension, or hyperlipidemia (all *p* for interaction > 0.05).

### 3.4. Sensitivity Analysis

We evaluated the robustness of our main findings through sensitivity analysis using an LMM. The results show that the inverted U-shaped association between SUA and HGS remained robust. Importantly, the sensitivity analyses revealed that the inverted U-shaped association between SUA and HGS remained equally significant in both the male and non-T2DM subgroups, which further supports the robustness of our main findings (Table 2).

## 4. Discussion

Based on a large community cohort with repeated measurements, we systematically investigated the longitudinal relationship between SUA and HGS and revealed three key findings. First, there was a significant nonlinear inverted U-shaped relationship between SUA and HGS, with an inflection point at 404 μmol/L. Below or at this threshold, SUA and HGS were positively correlated; above it, they were negatively correlated. Second, this inverted U-shaped relationship was significantly modified by sex and prominent in non-T2DM participants. Third, even after multiple model adjustments and sensitivity analyses, the inverted U-shaped relationship remained robust. These results suggest that SUA has a threshold effect on HGS that is based on gender and potentially related to specific glucose metabolism states.

One of the most important findings of this study is the existence of a nonlinear relationship between SUA and HGS, which is consistent with the overall trend of previous research, suggesting that there may be an optimal SUA concentration range beneficial to muscle function. For instance, Huang et al. [21] reported a significant inverted J-shaped association between SUA quartiles and muscle strength in a cross-sectional study of 630 Japanese men aged 30–83 years, with hyperuricemia patients showing notably lower muscle strength than non-hyperuricemia patients. Similarly, Xu et al. [20] found a significant inverted J-shaped association between SUA and HGS in a cross-sectional study of 992 Chinese individuals aged 45 and above, indicating protective effects in a SUA range. Liu et al. [29] performed a subgroup analysis of 4236 adults aged 50 years and older from communities in a cross-sectional study in Western China and identified a significant inverted J-shaped association between SUA and HGS in women. These studies confirm that a moderate SUA concentration range benefits muscle function and reflect a balanced SUA level where antioxidant benefits are maximized. The main contribution of our study is the precise quantification of the inflection point in this inverted U-shaped association at approximately 404 μmol/L in a large Chinese community cohort. This is close to China’s hyperuricemia diagnostic threshold (SUA level ≥ 420 μmol/L) [30], which indicates that the potential adverse effects of SUA on muscle function might begin near this clinical threshold. This finding has implications for clinical risk assessment and early intervention.

This study observed an inverted U-shaped association rather than an inverted J-shaped one, and this association was only significant in men and non-diabetic populations, which differs from some previous studies. These differences may be attributed to the following aspects. First, there is a fundamental difference in research design. Unlike the cross-sectional designs employed by most studies reporting inverted J-shaped associations, our study utilized a longitudinal design with repeated measurements. Cross-sectional studies capture the SUA–HGS relationship at a single time point, which may only reveal a fragment of the complete dose–response curve. In contrast, a repeated-measures cohort tracks changes in SUA levels and HGS over time. Beyond accounting for individual heterogeneity, this approach considers temporal trends, enabling a more comprehensive observation of the dose–response relationship. Furthermore, repeated measurements minimize the confounding effects of temporal biological variability and measurement error inherent in single assessments, thereby capturing the true nature of the SUA–HGS association more accurately. Consequently, previous studies demonstrating inverted J-shaped curves may have failed to fully capture the positive correlation between low SUA levels and HGS, observing only the trajectory associated with higher SUA concentrations. Secondly, the composition of the research population and the distribution of data might be another key reason for the curve differences in nonlinear relationships. If the SUA level of a cohort is mainly concentrated within the normal and high range, its analysis results may tend to show a negative correlation or an inverted J-shaped pattern. Conversely, if the SUA level of the study population is mainly concentrated in the low range, the analysis results may show a positive correlation. The cohort of this study may have covered a wider and more balanced horizontal distribution of SUA and thus may present a complete inverted U-shaped curve. Comorbidities in different study populations are also a key factor influencing HGS in addition to SUA. Finally, this study revealed that the inverted U-shaped relationship only exists in men, which is different from the inverted J-shaped association discovered in women in Western China [29]. This might reflect the complex role of sex hormones in SUA metabolism and muscle function regulation.

The potential mechanisms underlying the inverted U-shaped association between SUA and HGS in this study may relate to dual functions of SUA at different concentrations in muscle function. Within physiological concentration ranges, SUA, as one of the most important physiological antioxidants in plasma, may predominantly exert muscle-protective effects [31]. SUA can inhibit oxidative reactions through direct free radical scavenging and metal ion chelation, reducing oxidative stress from muscle tissue metabolism, thereby protecting myofiber structure and function and maintaining muscle strength [9,32]. Therefore, when SUA levels are excessively low, the body may lose this critical antioxidant barrier and become exposed to higher oxidative stress levels, leading to muscle protein damage and functional impairment, constituting the left ascending segment of our observed inverted U-shaped curve. However, high SUA levels exceeding physiological thresholds exhibit oxidative effects. High concentrations of SUA generate uric acid radicals through oxidation, activate enzyme systems including NADPH oxidase (NOX) and xanthine oxidase (XO), exacerbate excessive ROS production, and induce mitochondrial dysfunction. This affects Adenosine Triphosphate (ATP) generation and energy supply, collectively causing muscle tissue energy metabolism disorders and functional decline, thus providing a pathophysiological foundation for the right descending segment of the inverted U-shaped curve [33,34,35].

Potential mechanisms explaining why this study found an inverted U-shaped association between SUA and HGS in men may relate to sex hormone differences. Higher male testosterone levels inhibit uric acid excretion, leading to higher baseline SUA levels and causing the SUA distribution to more completely cover both sides of the inverted U-shaped curve [36,37]. In women, dramatic estrogen level changes before and after menopause affect SUA metabolism, with estrogen promoting urate excretion and inhibiting xanthine oxidase activity. This means female SUA levels are maintained in lower ranges in the long term, causing population data to fall mainly on the curve’s left side, making complete U-shapes or right-side negative associations difficult to observe [38]. Importantly, this study also reveals that potential mechanisms underlying the inverted U-shaped association between SUA and HGS may only exist in non-T2DM participants, likely because T2DM itself causes systemic chronic inflammation and significantly elevated oxidative stress states, masking SUA’s more refined regulatory effects on HGS [39,40,41]. In T2DM patients, the pathophysiological processes of diabetes rather than fluctuations in SUA levels may be dominant factors affecting HGS changes [42,43,44,45].

This study possesses several notable strengths. First, this study was based on a large community adult cohort and repeated-measures data, providing sufficient statistical power to reliably detect nonlinear relationships and conduct subgroup analyses. Second, employing an RCS methodology allowed us to flexibly capture nonlinear relationships while controlling for intra-individual correlations. Finally, subgroup analyses revealed effect heterogeneity and provide clues for potential future individualized interventions. Nevertheless, this study has several limitations. First, although we adjusted for common confounders such as age, BMI, hypertension, and hyperglycemia in our models, residual confounding remains a possibility. In particular, several key confounders that may simultaneously influence both SUA and HGS were not captured, including renal function indicators [46], dietary patterns and purine intake [47], medication history affecting uric acid metabolism [48,49], and biomarkers potentially associated with muscle damage [50]. These unmeasured confounders may have influenced the observed associations between SUA and HGS. Future studies should more systematically collect data on renal function, diet, medication use, and relevant biomarkers within prospective designs and perform more comprehensive confounding control analyses to validate the robustness of these findings. Second, regular physical activity was treated as a dichotomous variable in this study; however, it is a multidimensional factor, and variations in its frequency, intensity, and duration may differentially affect oxidative stress, purine metabolism, and muscle function. This simplified categorization may not adequately capture the complex relationships between physical activity, SUA levels, and HGS. Comprehensive data collection is warranted in future investigations to further elucidate these interactions. Third, participants were recruited through a mobile health (mHealth) application, which may have preferentially included individuals with higher health awareness and digital literacy, potentially limiting the generalizability of the findings to the broader population. However, this selection characteristic is unlikely to substantially affect the internal validity of the observed dose–response relationship between SUA and HGS, though it may influence population-level estimates. Future studies based on population-representative samples are warranted to further validate these findings. Fourth, the observational study design precludes the establishment of causality. Fifth, the absence of data on SUA metabolites or muscle damage markers limits mechanistic exploration. Future research should incorporate these biomarkers to elucidate the underlying biological pathways linking SUA and muscle function.

## 5. Conclusions

In conclusion, our study provides robust longitudinal cohort evidence for an inverted U-shaped relationship between SUA and HGS in Chinese community-dwelling adults. This association appears to be modulated by sex and notably observed in non-T2DM participants, underscoring the necessity of adopting stratified and individualized approaches when interpreting the relationship between SUA levels and musculoskeletal health. From a clinical perspective, both excessively low and high SUA levels may be detrimental to muscle function, suggesting that the optimal SUA range for preserving muscle function warrants further exploration. As an easily accessible biomarker, SUA may, after further validation, help in identifying individuals at accelerated risk of muscle decline. Future research should prioritize prospective cohort studies with rigorous control of confounding factors and employ causal inference methods, such as Mendelian randomization, to elucidate the causal relationship between SUA and muscle function. Additionally, to better clarify the underlying pathophysiological mechanisms, comprehensive assessments incorporating muscle mass, muscle quality, and inflammatory biomarkers are warranted. Multi-omics analyses of muscle tissue could further advance mechanistic understanding by identifying specific biomarkers and elucidating the underlying biological pathways. Finally, validating population-specific optimal SUA thresholds across different ethnic and age groups is essential for achieving personalized SUA management strategies aimed at promoting musculoskeletal health.

## Figures and Tables

**Table 1:** Characteristics of the total cohort and subcohorts stratified by sex.

Variables	Level	Female (*n* = 2045)	Male (*n* = 971)	*p*-Value
Age (years)		71.60 (8.80)	74.22 (8.70)	<0.001
Region, *n* (%)	Central	424 (20.7)	217 (22.3)	0.001
	East	768 (37.6)	303 (31.2)	
	North	324 (15.8)	141 (14.5)	
	Southwest	529 (25.9)	310 (31.9)	
Education, *n* (%)	primary school or below	320 (15.6)	104 (10.7)	<0.001
	junior high school	447 (21.9)	190 (19.6)	
	senior high school	542 (26.5)	211 (21.7)	
	college or above	736 (36.0)	466 (48.0)	
BMI (kg/m^2^)	/	24.20 (3.23)	24.02 (2.96)	0.131
Regular physical activity, *n* (%)	No	483 (23.6)	303 (31.2)	<0.001
	Yes	1562 (76.4)	668 (68.8)	
Hypertension, *n* (%)	No	1131 (55.3)	458 (47.2)	<0.001
	Yes	914 (44.7)	513 (52.8)	
T2DM, *n* (%)	No	1468 (71.8)	652 (67.1)	0.01
	Yes	577 (28.2)	319 (32.9)	
Hyperlipidemia, *n* (%)	No	1306 (63.9)	696 (71.7)	<0.001
	Yes	739 (36.1)	275 (28.3)	
Serum uric acid (µmol/L)	/	406.92 (109.16)	437.28 (112.60)	<0.001
Handgrip strength (kg)	/	32.38 (12.79)	38.25 (10.73)	<0.001

Data are shown as the mean (standard deviation) for continuous variables and *n* (%) for categorical variables. Abbreviations: BMI, body mass index; T2DM, type 2 diabetes mellitus.

**Table 2:** Sensitivity analyses for the association between serum uric acid and handgrip strength in various subgroups.

Groups	Uric Acid Level	Model 1		Model 2		Model 3	
		Beta (95%CI)	*p*	Beta (95%CI)	*p*	Beta (95%CI)	*p*
**Overall**	**All**	−0.21 (−0.56, 0.15)	0.256	−0.24 (−0.59, 0.12)	0.192	−0.23 (−0.58, 0.13)	0.216
	**≤404 μmol/L**	1.58 (0.48, 2.69)	0.005	1.55 (0.44, 2.66)	0.006	1.55 (0.44, 2.66)	0.006
	**>404 μmol/L**	−0.57 (−1.14, 0.01)	0.054	−0.57 (−1.14, 0.01)	0.053	−0.58 (−1.15, 0.00)	0.051
**Subgroup analyses**							
**Sex**							
**Female**	**All**	−0.23 (−0.77, 0.32)	0.411	−0.30 (−0.85, 0.24)	0.275	−0.28 (−0.83, 0.27)	0.320
	**≤404 μmol/L**	0.76 (−1.17, 2.70)	0.438	0.71 (−1.25, 2.67)	0.477	0.68 (−1.28, 2.64)	0.496
	**>404 μmol/L**	0.19 (−0.64, 1.02)	0.650	0.19 (−0.64, 1.01)	0.655	0.19 (−0.63, 1.02)	0.648
**Male**	**All**	−0.25 (−0.70, 0.20)	0.281	−0.26 (−0.72, 0.19)	0.254	−0.26 (−0.71, 0.20)	0.272
	**≤404 μmol/L**	1.92 (0.59, 3.24)	0.005	1.90 (0.57, 3.22)	0.005	1.89 (0.56, 3.21)	0.005
	**>404 μmol/L**	−1.09 (−1.87, −0.32)	0.006	−1.10 (−1.88, −0.32)	0.006	−1.10 (−1.88, −0.32)	0.006
**T2DM**							
**No**	**All**	−0.28 (−0.69, 0.12)	0.166	−0.30 (−0.71, 0.10)	0.141	−0.31 (−0.71, 0.10)	0.136
	**≤404 μmol/L**	1.51 (0.28, 2.74)	0.016	1.48 (0.24, 2.72)	0.019	1.42 (0.19, 2.66)	0.024
	**>404 μmol/L**	−0.67 (−1.31, −0.02)	0.043	−0.66 (−1.30, −0.01)	0.047	−0.66 (−1.31, −0.01)	0.046
**Yes**	**All**	0.01 (−0.71, 0.72)	0.981	−0.06 (−0.78, 0.66)	0.863	−0.09 (−0.82, 0.63)	0.799
	**≤404 μmol/L**	1.73 (−0.65, 4.11)	0.154	1.62 (−0.77, 4.02)	0.184	1.67 (−0.73, 4.08)	0.173
	**>404 μmol/L**	−0.38 (−1.55, 0.78)	0.520	−0.36 (−1.52, 0.81)	0.549	−0.41 (−1.58, 0.76)	0.491
**Hypertension**							
**No**	**All**	−0.23 (−0.74, 0.29)	0.391	−0.28 (−0.80, 0.24)	0.285	−0.29 (−0.81, 0.23)	0.277
	**≤404 μmol/L**	2.09 (0.52, 3.65)	0.009	2.05 (0.48, 3.63)	0.011	1.99 (0.42, 3.57)	0.013
	**>404 μmol/L**	0.01 (−0.73, 0.74)	0.987	0.03 (−0.70, 0.76)	0.939	0.01 (−0.72, 0.74)	0.981
**Yes**	**All**	−0.18 (−0.67, 0.31)	0.478	−0.15 (−0.64, 0.34)	0.547	−0.17 (−0.66, 0.32)	0.501
	**≤404 μmol/L**	1.21 (−0.29, 2.70)	0.113	1.19 (−0.30, 2.69)	0.119	1.13 (−0.37, 2.62)	0.140
	**>404 μmol/L**	−1.11 (−1.99, −0.22)	0.014	−1.14 (−2.02, −0.25)	0.012	−1.15 (−2.03, −0.26)	0.012
**Hyperlipidemia**							
**No**	**All**	−0.06 (−0.48, 0.36)	0.770	−0.08 (−0.50, 0.35)	0.725	−0.02 (−0.45, 0.40)	0.909
	**≤404 μmol/L**	1.36 (0.13, 2.59)	0.030	1.38 (0.15, 2.62)	0.028	1.43 (0.20, 2.66)	0.023
	**>404 μmol/L**	−0.34 (−1.04, 0.36)	0.342	−0.32 (−1.02, 0.39)	0.380	−0.31 (−1.02, 0.39)	0.384
**Yes**	**All**	−0.30 (−0.95, 0.35)	0.370	−0.33 (−0.98, 0.32)	0.323	−0.32 (−0.97, 0.33)	0.337
	**≤404 μmol/L**	1.83 (−0.47, 4.14)	0.120	1.61 (−0.71, 3.93)	0.175	1.61 (−0.72, 3.93)	0.176
	**>404 μmol/L**	−0.67 (−1.65, 0.31)	0.179	−0.60 (−1.58, 0.38)	0.229	−0.60 (−1.59, 0.38)	0.228

Data are presented as beta coefficients (β) with their 95% confidence intervals (CIs) from linear mixed-effects models. The association was assessed in the overall population and stratified by sex, T2DM, hypertension, and hyperlipidemia status. Models were sequentially adjusted as follows: Model 1 was adjusted for age, sex, and region; Model 2 was additionally adjusted for BMI, educational level, and regular physical activity; Model 3, the fully adjusted model, was further adjusted for the prevalence of T2DM, hypertension, and hyperlipidemia. Statistically significant associations (*p* < 0.05) are highlighted in bold. Abbreviations: CI, confidence interval; T2DM, type 2 diabetes mellitus; BMI, body mass index.

## Data Availability

The datasets generated and/or analyzed during the current study are not publicly available due to privacy restrictions but are available from the corresponding author upon request.
